# Implications of neuroimaging findings in addiction

**DOI:** 10.1093/psyrad/kkad006

**Published:** 2023-04-29

**Authors:** Xinwen Wen, Lirong Yue, Zhe Du, Linling Li, Yuanqiang Zhu, Dahua Yu, Kai Yuan

**Affiliations:** School of Life Science and Technology, Xidian University, Xi'an 710126, China; School of Life Science and Technology, Xidian University, Xi'an 710126, China; School of Life Science and Technology, Xidian University, Xi'an 710126, China; School of Biomedical Engineering, Medical School, Shenzhen University, Shenzhen 518060, China; Guangdong Provincial Key Laboratory of Biomedical Measurements and Ultrasound Imaging, Shenzhen University, Shenzhen 518060, China; Department of Radiology, Xijing Hospital, Fourth Military Medical University, Xi'an 710032, China; Inner Mongolia Key Laboratory of Pattern Recognition and Intelligent Image Processing, School of Information Engineering, Inner Mongolia University of Science and Technology, Baotou 014010, China; School of Life Science and Technology, Xidian University, Xi'an 710126, China; Engineering Research Center of Molecular and Neuro Imaging Ministry of Education, Xidian University, Xi'an 710126, China; Xi'an Key Laboratory of Intelligent Sensing and Regulation of trans-Scale Life Information, School of Life Science and Technology, Xidian University, Xi'an 710126, China

Addiction is a chronic and often relapsing brain disorder characterized by drug abuse and withdrawal symptoms and compulsive drug seeking (Koob and Volkow, 2010) when access to the drug is restricted. Addiction leads to structural and functional brain changes implicated in reward, memory, motivation, and control (Volkow *et al*., 2019; Lüscher *et al*., 2020). Neuroimaging technology provides us with the scientific and intuitive methods (Canario *et al*., 2021; Dong *et al*., 2022) to study brain mechanism of addiction noninvasively, which has built a bridge between addictive behavior and neural mechanism. Brain structural and functional plasticity has been studied in addiction, such as dysfunctions in the dopaminergic system (Liu and Li, 2018) and corticostriatal circuits (Volkow and Morales, 2015; von Deneen *et al*., 2022). Notably, the brain activation and connectivity patterns in abstainers exposed to drug cues and their associations with future relapse risk were assessed using functional imaging (Grüsser *et al*., 2004; Beck *et al*., 2012). In addition, neuroimaging has been used to predict the relapse risk in drug-dependent individuals following unsuccessful treatment and to identify brain biomarkers (Yip *et al*., 2020) that are relevant to the pathology and repeated transcranial magnetic stimulation (rTMS) treatment of addiction. Moreover, the neural mechanisms of efficacy following rTMS treatment (Jin *et al*., 2022) were investigated systematically from regional and circuits levels. However, the high relapse rates of addiction remain an urgent challenge to be addressed in further research.

## Dysfunction of the Brain Dopamine System in Addiction

The formation of heroin, methamphetamine, nicotine, and other addictions is related to the intense reward, and the brain dopamine (DA) system is considered to be an important component of the reward process in addiction (Volkow and Morales, 2015; Volkow *et al*., 2019). With the development of PET and SPECT techniques, the noninvasive research of brain metabolism, receptors, and neurotransmitters makes it possible to study the molecular mechanism of reward in drug-dependent individuals. The dopaminergic dysfunctions, i.e. reduced DA release, DA D2 receptors, and DA transporters (DAT) in bilateral striatum (Volkow *et al*., 2007; Kim *et al*., 2011; Ashok *et al*., 2017; Peters *et al*., 2021), have been described in drug-dependent individuals, and long-term abstinence was beneficial to the recovery of impaired brain DA neurons (Shi *et al*., 2008). Similarly, prolonged use of methamphetamine might result in severe symptoms of addiction and the reduction of DAT density in DA neurons in the brain (Sekine *et al*., 2001; Witt *et al*., 2020). These findings in the DA system encouraged us to explore the association between DA-related regions and other brain regions as well as these roles in addictive behaviors.

The alterations in neurotransmitter systems, which include not only the DA system but also glutamate, serotonin, γ-aminobutyric acid (GABA), and endocannabinoids, have been involved in addiction. Drugs of abuse have negative effects on executive function circuits, reward circuits, and stress circuits via these neurotransmitter-specific activities at the ventral tegmental area or nucleus accumbens. Dysregulation of glutamatergic, GABAergic, and stress-related neuronal activity triggered by uncontrolled drug administration drive deficits in neurocircuitry associated with executive function and might mediate vulnerability, perpetuation, and relapse of addiction.

## The Corticostriatal Circuits Plasticity Changes in Addiction

Different types of drug addiction showed similar abnormal structural characteristics in the striatum, prefrontal cortex, hippocampus, and other brain regions, and their functional activation in craving and cognitive tasks were also different from healthy controls (Bi *et al*., 2017; Liu *et al*., 2019; Yang *et al*., 2021). These neuroimaging findings might be related to the reward deficit and impaired cognitive behavior of addicted individuals. Addiction results in supraphysiologic surges of DA in the NAc to activate the striatal-related DA pathway directly and inhibit the striatal-cortical pathway indirectly, indicating that drugs influence not only the local alteration of these brain regions but also the interaction of multiple brain circuits (Volkow and Morales, 2015). The abnormalities in structural and functional connectivity were also found in several brain circuits containing corticostriatal circuits, midbrain pathways, and thalamic-cortical circuits involved in reward processing, cognitive control, and craving (Yuan *et al*., 2017; Yuan *et al*., 2018). The critical role of corticostriatal circuits in addiction has become the primary focus associated with reward in the striatum and cognitive control in the prefrontal cortex (PFC) (Yuan *et al*., 2016; Yuan *et al*., 2017; Yuan *et al*., 2018; Yuan *et al*., 2018; Liu *et al*., 2021; Lu *et al*., 2022). The circuits involve parallel loops including different pathways from different areas of the PFC to different subregions of the striatum, implicating cognitive, impulsivity, compulsivity, and reward processing in addiction behavior (Robbins *et al*., 2012). Impulsivity traits and dysfunctional reward circuits of ventral- and dorsal striatum in drug-independent individuals may lead to a propensity toward drug abuse. Both impaired structural and functional connectivity in ventral and dorsal striatum-PFC circuits were revealed in heroin users (Lu *et al*., 2022), which complements neuroimaging evidence for the dramatic dysregulation of key circuits involved in craving, impaired inhibitory control, and reward deficits in addiction (Everitt and Robbins, 2013; Koob and Volkow, 2016). Although there are similar changes in corticostriatal circuits, the ventral striatal-frontal circuits implicated in incentive salience and the dorsal striatal-frontal circuits associated with adaptive regulation of a drug-use habit show distinct functions in the transition and formation of addiction (Vollstädt-Klein *et al*., 2010; Zhou *et al*., 2018; Gerchen *et al*., 2019; Zhou *et al*., 2019; Ersche *et al*., 2020). The dysfunction of the midbrain-striatal circuit involved in dopaminergic modulation and the midbrain-cortical circuit linking to inhibition control discovered in drug abusers demonstrated the central roles of midbrain pathways in reward processing of addiction (Xu *et al*., 2021). The thalamus plays an indispensable role in the reward process and response inhibition as a critical component of the cortico-striato-thalamo-cortical loop in addiction (Huang *et al*., 2018). The altered functional connectivity of thalamic subregions-cortical circuits involved in the reward and withdrawal symptoms was shown in drug abusers (Zhang *et al*., 2021). Similar neuroplastic changes including altered gray matter volume and connectivity of striatal regions and circuits involved in reward and cognitive control were also detected for internet gaming disorder (IGD) (Qin *et al*., 2020; Zhou *et al*., 2020; von Deneen *et al*., 2022; Yu *et al*., 2022). IGD has been recently included as a mental disorder in the ICD-11 and DSM-V (Grant and Chamberlain, 2016). However, there is still an ongoing debate on the differences and similar striatal alterations between drug addiction and IGD. In a neuroimaging-based meta-analysis, the distinct striatal activation patterns in drug addiction and behavioral addiction during reward outcome were reported to be relevant to reward deficiency (Luijten *et al*., 2017). These cross-sectional studies suggested that repeated drug administration or behavior triggered neuroplastic changes in several critical brain circuits. Future explorations would also focus on clarifying the neural patterns of different addiction types and obtaining the specific biomarkers for addiction treatment.

## Brain Recovery after Prolonged Abstinence in Addiction

It is worth noting that the phenomenon of brain recovery induced by prolonged abstinence has triggered researchers’ attention. Previous longitudinal studies in methamphetamine addiction observed the recovery of DA function in the striatum after prolonged abstinence. We also found that the cortical thickness in prefrontal regions in heroin and cocaine addiction users tended to be normal accompanied by decreased craving scores after prolonged abstinence (He *et al*., 2018; Yang *et al*., 2022). The increased functional connectivity of the midbrain-cortical circuits shown after 10 months' abstinence indicated partial brain recovery of impaired function during abstinence (Xu *et al*., 2021). Furthermore, the brain structure in Fractional Anisotropy (FA) values and functional connectivity in the frontal-striatal circuits were correlated to craving changes and cognitive scores observed in heroin abstainers (Lu *et al*., 2022), which means that these circuits might be potential biomarkers for cognition and craving changes. From the dynamic perspective, reconfigurations of dynamic functional network connectivity in the large-scale brain network were also observed, which improved the understanding of the neurobiology of prolonged abstinence in heroin users (Zhang *et al*., 2022). These findings have significant social benefits by providing scientific evidence for addiction treatment and enhancing the confidence of addicted patients, their families and society.

## Multimodal Neuroimaging Predicts Relapse after Abstinence

Addiction relapse rates following abstinence or other treatment remain high, though evidence-based treatments have progressed (Carroll and Onken, 2005; Dutra *et al*., 2008). Early treatment is critical and a vulnerable period for future relapse (Connery, 2015). How to successfully predict relapse is critical to promote recovery and neurobiological models of addiction. Most studies used unimodal neuroimaging to predict craving or relapse (Yuan *et al*., 2018; Zhao *et al*., 2020; Wen *et al*., 2021), suggesting that this work could offer promising and alternative approaches to enhance the prognostic and predictive ability of treatment response. The functional network implicated in cognitive/executive control and in reward responsiveness predicted abstinence during treatment with 64% accuracy (Yip *et al*., 2019). Brain structural tract connecting the anterior insula to nucleus accumbens (NAc) predicts relapse to stimulant drug use after abstinence and suggests that the specific insular structural connectivity to NAc may be a biomarker for relapse risk (Tisdall *et al*., 2022). Both functional and structural connectivity of dorsolateral PFC-circuits were able to predict drug cue-induced craving changes and relapse in abstainers (Liu *et al*., 2021). However, these studies focused on exploring potential neuroimaging markers and were limited to prediction accuracy and the likelihood of reproducibility in novel datasets due to the overfitting data and restricted brain features (Yip *et al*., 2020; Li *et al*., 2021). Increasing multimodal neuroimaging studies demonstrated that combining all modalities could improve the performance of individualized prediction in psychiatry (Ooi *et al*., 2022). Statistically, the predictive potential of multimodal neuroimaging representing multi-dimensional brain information was certified to be significantly higher than the performance of unimodality (Sui *et al*., 2020). Using a longitudinal design, multimodal neuroimaging data with machine learning suggested that it could accurately predict alcohol use disorder remission (Kinreich *et al*., 2021). In conclusion, the generalizability of methods to identify individuals at relapse risk for unsuccessful treatment and promoting agreement between predicted and observed clinical outcomes will be enhanced by combining machine learning with multimodal neuroimaging. If relapse risk following treatment can be foreseen at an individual level, the individualized treatment strategy will be improved to reduce the relapse rate and potential death risk. More efforts are needed to translate existing models into clinical application, develop prognostic models for early prevention, and inform clinical decisions on addiction treatment.

## Noninvasive Brain Stimulation for Addiction Treatment

Repeated transcranial magnetic stimulation (rTMS) has been proved to be effective (Zhang *et al*., 2019; Yuan *et al*., 2020) in treating different drug addictions (heroin, nicotine, alcohol) through noninvasive stimulation of specific brain cortical functions such as bilateral PFC. 1-Hz rTMS and 10-Hz rTMS were both effective in reducing the cue-induced craving in heroin users and the effects were lasting after treatment (Liu *et al*., 2020). Moreover, deep rTMS decreased alcohol craving following treatment and the percentage of heavy drinking days during follow-up (Harel *et al*., 2022). Besides, the neural mechanism underlying the efficacy of rTMS intervention in addiction has been revealed using neuroimaging techniques, which mainly included coupling of the corticostriatal and insula circuits (Jin *et al*., 2022), changes in functional connectivity between brain networks, and alteration in the level of GABA (Su *et al*., 2020). These studies mostly used the “5-cm method” for target localization of the stimulation in the left prefrontal cortex. The efficacy of this therapeutic intervention might largely depend on the positioning of stimulation target regions (Cash *et al*., 2021). Converging evidence has demonstrated the poor targeting accuracy of the “5-cm method” due to inter-subject variability, providing clear support for the need for more accurate localization techniques for target regions. Compared with the traditional rTMS technique, neuronavigation enables the rTMS target to be accurately positioned to specific sites based on individual anatomical imaging or functional activation regions (Cash *et al*., 2021; Cole *et al*., 2022), which may improve the intervention effect because the technique is less susceptible to inter-subject variability and has shown great advantages in depression interventions. Thus, the fusion of activation site in the frontal cortex during cue-induced craving and neuronavigation rTMS may offer a promising strategy for addiction intervention by achieving more scientific, accurate, and effective target localization, ultimately enhancing intervention outcomes. The intervention effect of noninvasive brain stimulation using open-loop methods has been largely related to individual state. Closed-loop neuromodulation , a new noninvasive neuromodulation technique, intervenes in individual targets or circuits temporally for personalization when the specific state is detected. This approach can more precisely and efficiently regulate characteristic pathological lesions to achieve a better therapeutic effect, which has been proved in the treatment of depression (Scangos *et al*., 2021).

## The Challenges of Neuroimaging Technology in Addiction

Based on the above neuroimaging literature, we further propose: (1) The integration of multimodal neuroimaging has the potential to extend our understanding of neuroplasticity in addiction; (2) Multimodal neuroimaging combined with a cross-validation predictive model may enhance prediction of relapse risk and the likelihood of reproducibility in novel independent individuals; (3) Neuronavigation rTMS provides an advanced strategy to improve the intervention effect in addiction treatment by positioning the stimulation target accurately. In sum, combining artificial intelligence with noninvasive brain stimulation, neuroimaging technology will provide more effective guidance for addiction treatment.

**Figure 1:**
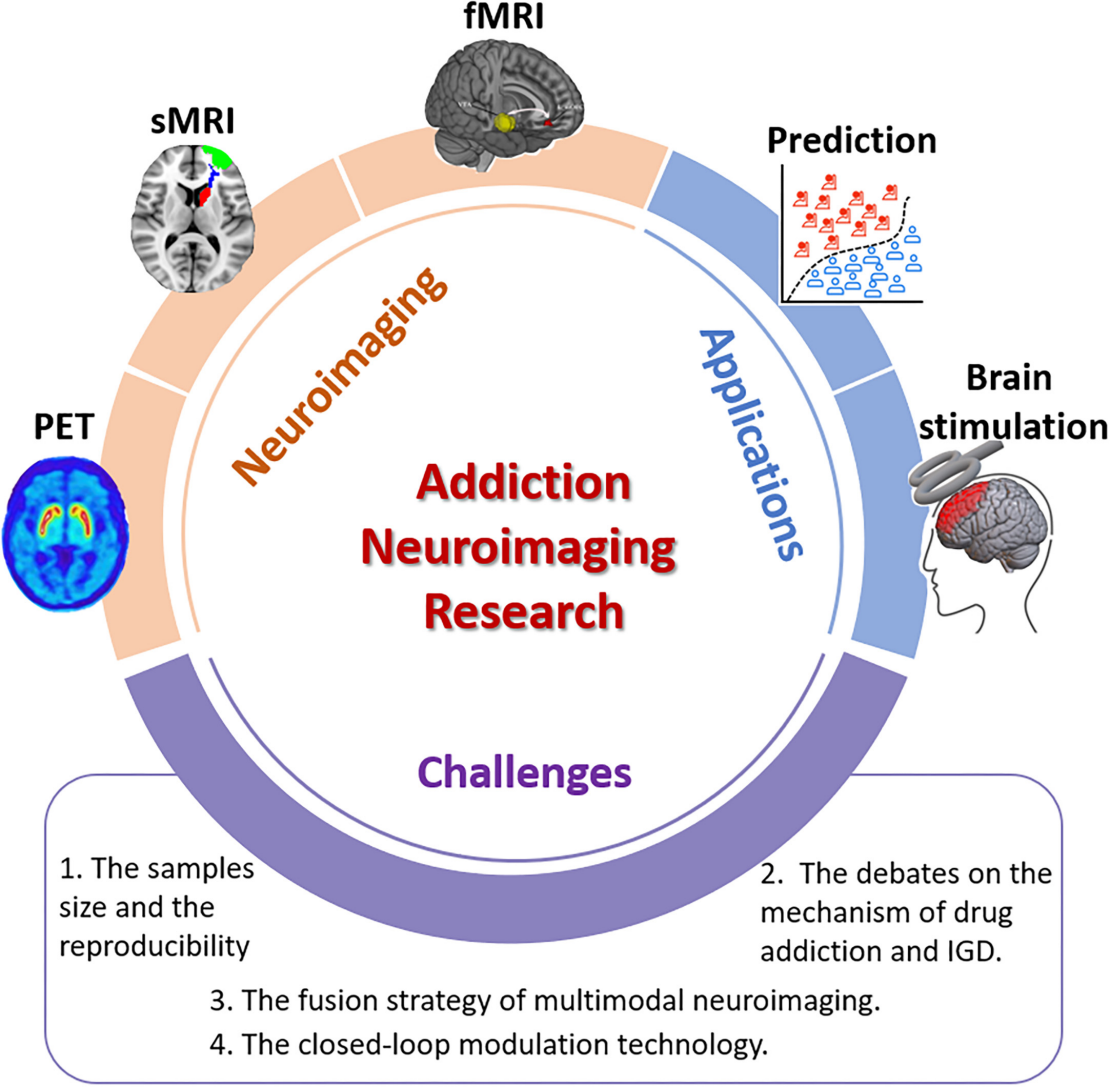
This perspective of addiction neuroimaging research includes brain plasticity using different neuroimaging techniques, its application in relapse prediction or brain stimulation, and the urgent challenges to be addressed in the future. IGD: internet gaming disorder.

There are several urgent challenges in applying neuroimaging technology to addiction research (Fig. 1). (1) Sample sizes and the reproducibility of previous findings and standardization between different devices. The establishment of open and sharing databases such as the Human Connectome Project and the Adolescent Brain Cognitive Development (ABCD) study, and the development of analysis methods provide approaches to solve this challenge. Therefore, building broad and shared neuroimaging datasets of addiction is an important future direction to increase the reproducibility likelihood in novel clinical samples of existing neuroimaging evidence and to achieve more robust prediction models of relapse risk or onset age of addiction, benefiting the understanding of addiction mechanism and clinical guidance on addiction treatment. (2) Characterizing the longitudinal trajectories of brain development that contribute to periods of addiction vulnerability is important, thus it is necessary to incorporate the evidence of brain development and the neuroimaging findings of brain functions in addiction (Bogdan *et al*., 2023). (3) Different types of addiction have different neural patterns that may be considered as specific targets in addiction intervention. Integrating existing neuroimaging findings across different types of addiction can help optimize treatment guidance for patients according to type of addiction. (4) How to achieve individualized diagnosis and treatment. When using traditional noninvasive brain stimulation techniques, individual differences in clinical effect still exist. Recently, closed-loop modulation technology, an adaptive intervention strategy based on real-time status, has been shown to be more effective in clinical studies (Sitaram *et al*., 2017; Basu *et al*., 2021; Scangos *et al*., 2021), providing a novel insight for individualized addiction treatment. The progress in neuroimaging findings and therapy could potentially be transferred cautiously to the treatment of gaming disorder in the future.
